# Exploring the Effects of Different Bacteria Additives on Fermentation Quality, Microbial Community and In Vitro Gas Production of Forage Oat Silage

**DOI:** 10.3390/ani12091122

**Published:** 2022-04-27

**Authors:** Yi Xiong, Jingjing Xu, Linna Guo, Fei Chen, Dedai Jiang, Yanli Lin, Chunze Guo, Xiaomei Li, Yunrong Chen, Kuikui Ni, Fuyu Yang

**Affiliations:** 1 College of Grassland Science and Technology, China Agricultural University, Beijing 100193, China; xiongleslie@126.com (Y.X.); xucau@outlook.com (J.X.); linnam921@163.com (L.G.); 17683267025@189.cn (F.C.); sy20203243207@cau.edu.cn (D.J.); guocz19970404@163.com (C.G.); b20193040360@cau.edu.cn (X.L.); nikk@cau.edu.cn (K.N.); 2 Beijing Sure Academy of Biosciences, Beijing 100193, China; lyl161@126.com; 3 Donghan Animal Husbandry and Veterinary Station of Fuqing City, Fujian 350300, China; 15005959791@163.com

**Keywords:** forage oat, lactic acid bacteria, *Propionibacterium*, fermentation quality, greenhouse gas

## Abstract

**Simple Summary:**

Forage oat is an important feed resource in the world. Few studies on the application of different bacterial additives in forage oat silage have been found, which limits the utilization and promotion of oat silage in animal husbandry. In this study, we compared the fermentation quality and in vitro gas production of oat silage treated with four additives (*Lactiplantibacillus* *plantarum* F1,LP; *Lacticaseibacillus* *rhamnosus* XJJ01, LR; *Lacticaseibacillus* *paracasei* XJJ02, LC; and *Propionibacterium acidipropionici* 1.1161, PP). The results show that compared to the CK group (without additives), the LR group had a higher dry matter content, while the LP group showed an improvement in fermentation quality. At the same time, the bacterial community in the LR group was also different from that in other groups. The treatments of PP and LC had no significant effects on fermentation quality, but the in vitro gas production was significantly reduced in the treated oat silage. These results could help us to optimize the utilization of forage oat silage in balanced ruminant diets.

**Abstract:**

Bacterial inoculants are considered as a good choice for successful ensiling, playing a key role in improving the silage quality. However, the potential of different bacteria, especially the propionic acid bacteria, in forage oat ensiling is yet to be explored. Therefore, the purpose of this study was to investigate the regulation effects of different bacterial additives on the fermentation quality of forage oat silage. Four additives (*Lactiplantibacillus plantarum* F1, LP; *Lacticaseibacillus 0rhamnosus* XJJ01, LR; *Lacticaseibacillus paracasei* XJJ02, LC; and *Propionibacterium acidipropionici* 1.1161, PP; without additives, CK) were inoculated in forage oat silage, and the fermentation quality and organic compounds were determined after 60 days of ensiling. Notably, LR showed higher dry matter preservation compared to other additives and CK. In addition, LP and LR showed strong lactic acid synthesis capacity, resulting in lower pH compared to other additives and CK. The treatments of PP and LC increased the bacterial diversity in silage, while the bacterial community in the LR group was different from that in other groups. In addition, the PP- and LC-treated oat silage showed significantly lower total in vitro gas production and a lower methane content. These results suggest that LP is more favorable for producing high-quality oat silage than LR, LC, or PP. Both the PP- and LC- treated oat silage may reduce rumen greenhouse gas emissions.

## 1. Introduction

Forage oat (*Avena sativa*) silage is considered a palatable, highly digestible and beneficial forage for ruminants in most parts of the world [1,2]. Large amounts of forage are consumed by ruminants, and the global trade of oat has been restricted due to the COVID-19 pandemic [3]. Meanwhile, oats have been widely cultivated in China, both as silage and hay, especially in northern China. In recent years, the research on oat silage has received continuous attention. In previous studies, low-temperature-tolerant lactic acid bacteria (LAB) and bacterial diversity in oat silage have been systematically studied [4,5]. However, different bacterial species in oats still need further exploration.

LAB plays an active role in silage processing and is widely used in various grass-based silages to reduce nutrient loss and extend the storage period. In addition, exogenous probiotics can normally change the type of fermentation in oat silage [6]. Therefore, adding exogenous bacteria or microbiota to silage has become an important way to regulate its anaerobic fermentation, and it also has a profound impact on the organic acid content and dry matter loss of oat silage [7]. *Propionibacterium* has been used as a microbial additive for forage oat to improve the silage quality [8]. In some cases, *Propionibacterium* provides better aerobic stability [9]. Silage inoculated with *Propionibacterium* can produce propionic acid in the anaerobic fermentation period to improve the aerobic stability [9]. However, there are still few reported studies on fermentation quality and in vitro gas production of oat silage treated with *Propionibacterium*.

Silage treated with lactic acid bacterial inoculants has been reported to increase ruminal microbial biomass when tested in vitro [10]. The rumen is one of the most powerful fiber-degrading fermentation systems known to date [11]. However, the degradation of fibers by a large number of microorganisms in the rumen produces organic acids, along with a large amount of methane and hydrogen. Methane (CH_4_) is a potent greenhouse gas and, along with carbon dioxide and nitrous oxide, CH_4_ emission from livestock production is a major contributor to global warming [12]. It is well-established that factors such as nutrient composition and degradability of ruminant diets greatly affect CH_4_ production [13]. In the in vitro experiment, *Propionibacterium freudenreichii* 53-W was found to exhibit the ability to reduce methane, but the relevant mechanism is not yet clear [14]. We hypothesize that silage treated with lactic acid bacteria or *Propionibacterium* can not only improve fermentation quality but also reduce methane emissions.

Therefore, this study aimed to investigate the effects of three different strains of LAB and one *Propionibacterium* strain on the fermentation quality of oat silage and in vitro gas production.

## 2. Materials and Methods

### 2.1. Additives and Ensiling

The four bacterial additives used in this study were Lactiplantibacillus plantarum F1 (isolated by Guo [15]), Lacticaseibacillus rhamnosus XJJ01, Lacticaseibacillus paracasei XJJ02 and Propionibacterium acidipropionici 1.1161. L. rhamnosus XJJ01 and L. paracasei XJJ02 (Accession Number: OK021555, OK021556) were isolated from cheese and stored in our laboratory. P. acidipropionici 1.1161 was purchased from Guangdong Microbial Culture Collection Center.

The oat forages were harvested in Kangbao County, Zhangjiakou City, Hebei Province, China (114°11′–114°56′ E, 41°25′–42°08′ N), which has an average altitude of 1450 m and an average annual temperature of 1.2 °C. On 3 October 2020, the fresh forage oat was harvested at the early milk stage and then directly chopped into segments at a theoretical length of 20 mm. Five hundred grams of fresh oat forages was stored in a cryogenic storage box and quickly brought back to the laboratory for raw materials analysis. The chemical composition of the oat forage is shown in Table 1. The control group was treated with no additives (CK), and the other four groups were treated with *L. plantarum* F1 (LP), *L. rhamnosus* XJJ01 (LR), *L. paracasei* XJJ02 (LC) and *P. acidipropionici* 1.1161 (PP) at 1.0 × 10^6^ colony-forming units (CFU)/g of fresh matter (FM). Approximately 300 g of chopped oat was packed in polyethylene bags (18 × 26 cm), which were vacuum-sealed. A total of 15 bags (3 replicates) were prepared and equally distributed and stored in ambient temperature for 60 days.

### 2.2. Analysis of Chemical Composition and Fermentation Quality

The samples of fresh forages and silage were collected for analysis after sealing in polyethylene bags. About 200 g of each group of fresh material and silage sample was dried in an air-drying oven at 65 °C for 48 h to measure dry matter (DM) content and then milled by a hammer crusher before passing through a 0.425 mm screen for chemical composition determination and in vitro ruminal fermentation. Crude protein (CP) and EE (ether extract) were analyzed according to AOAC [15]. The content of neutral detergent fiber (aNDF) and acid detergent fiber (ADF) was assayed using the method of Van Soest [16], quantifying aNDF while adding heat-stable α-amylase to the Ankom A2000i system (ANKOM Technology, Macedon, NY, USA). Water-soluble carbohydrate (WSC) content was assayed using the anthrone method [17].

The silage samples (20 g) were mixed with 180 mL of sterilized water, stored at 4 °C for 24 h and then serially diluted from 10^−1^ to 10^−5^. Then, 20 μL of each dilution at 10^−1^, 10^−3^ and 10^−5^ was spread onto the corresponding agar separately. The microbial populations were measured as described in our previously study [4]. The 10^−1^ diluted samples were filtered through a 0.22 μm filter, and the pH value was measured by a pH meter (PHS- 3C, INESA Scientific Instrument, Shanghai, China). The filtrate was used to determine the ammonia nitrogen (NH_3_-N) and organic acids. The NH_3_-N content was determined by the phenol hypochloric acid colorimetry method [18]. Lactic acids (LA), AA (acetic acids), PA (propionic acids) and BA (butyric acids) were determined via high-performance liquid chromatography (HPLC) as described in our previously study [4]. The fermentation quality of the oat silage was evaluated by Flieg’s point index, which was calculated as follows [19,20]:

Flieg’s point = 220 + [(2 × %DM) × 15] − 40 × pH(1)

### 2.3. Analysis of Microbial Community

Ten-gram samples were immediately put into 90 mL of sterile water for collecting microorganisms and treated with a shaker at 160 rpm for 2 h (4 °C). Then, they were filtered through two layers of sterile gauze and rinsed for several times with sterile water to recover residual microorganisms. The filtrates were centrifuged at 10,000× *g*, 4 °C, for 15 min. Total bacterial DNA was extracted from each sample of the oat silage according to the method described in a previous study [16].

The total DNA was amplified with the bacterial 16 S rDNA primers targeting the V3-V4 regions of 338F (ACTCCTACGGGAGGCAGCAG) and 806R (GGACTACHVGGGTWTCTAAT). The PCR conditions, sequencing and analyzing details conformed to the protocol described by Guo [15]. The diversity index and microbial community of the oat silage were calculated via bioinformatics cloud analytics platform (https://cloud.majorbio.com/. accessed on 15 January 2022).

### 2.4. Gas Production of Oat Silages

The ruminal fluid was obtained from three ruminally fistulated small-tail Han sheep (corn–soybean meal diet with alfalfa hay) before morning feeding. It was then immediately taken to the laboratory, filtered through four layers of gauze, kept at 39 °C in a water bath and blended with buffer solution (1:2, *v*/*v*) under a continuous flush of CO_2_ [17]. Two hundred milligrams of the samples were put into a 100 mL glass syringe with 30 mL of the mixed solution added in. After the addition of the liquid, it was placed in an artificial rumen incubator in a 39 °C water bath. There were three replicates for each sample, and three blank controls were set up. Then, the amount of gas generated after 0 h, 2 h, 4 h, 6 h, 8 h, 12 h, 24 h, 36 h, 48 h, 60 h and 72 h was recorded, and finally, the gas was collected and its composition was determined by gas chromatography. The temperature of both the thermal conductivity detectors and the oven was 100 °C. Standard gas, CH_4_, was used at a concentration of 25.0%; CO_2_ at 65.0%; H_2_ at 2.03% and O_2_ at 2.00%. The balance gas was nitrogen, and carrier gas was high-purity argon. The flow rate was 30 mL/min. The pressure was 0.5 MPa; and the volume of each injection was 1 mL.

### 2.5. Statistical Analysis

All the data were analyzed using one-way analysis of variance (ANOVA) to determine the variables of different inoculant treatments, and post hoc analysis was performed using Duncan’s multiple range test with SPSS 24.0 software (IBM Corp., Armonk, NY, USA). After gas production at different time points was calculated, the in vitro fermentation parameters, such as the potential gas production (B) and gas production rate constant (c), were calculated via SAS 9.3 software (SAS Institute Inc., Cary, NC, USA) NLIN program. Statistically significant difference was set at *p* < 0.05 and *p* < 0.01.

## 3. Results

### 3.1. Effects of Inoculants on the Chemical Composition and Fermentation Quality

Table 2 shows the chemical composition and microbial population by plate culture after 60 d of ensiling. Though the four inoculant treatments did not show significant differences in the contents of CP, EE, NDF or ADF, significant effects on the contents of DM and WSC were observed. The LR-treated oat silage had a higher DM content than the other inoculants and CK (*p* < 0.01). The LC-treated oat silage showed a higher WSC content than CK and PP, but there was no significant difference among LP, LC and LR treatments. The amount of LAB was higher in the LR-treated silage than the other inoculants and CK (*p* < 0.05).

The fermentation quality of the oat silage is shown in Table 3. LP had a lower pH than PP, LC and CK (*p* < 0.05), while the pH of LR was intermediate and not different from either LP or CK. (*p* > 0.05). In addition, LP had a lower NH_3_-N content than all the other treatments (*p* < 0.05). LR had a higher LA content than PP, LC and CK (*p* < 0.05). In addition, there were no significant differences in LA content among CK, LP, PP or LC (*p* > 0.05). Both LP and LC had a lower BA content than CK and LC (*p* < 0.05) but no significant difference from PP. Overall, LP had a higher Flieg’s point than CK, PP and LR.

### 3.2. Bacterial Community Analysis of the Oat Silage

As shown in Table 4, compared to CK, there was no significant difference among any of the treatments (*p* > 0.05), but the Shannon index was higher in the oat silage in PP and LC than in other inoculants (*p* < 0.05). None of the treatments had significant effects on the Simpson, ACE or Chao indices. In addition, all coverage values were higher than 0.999, which indicates that the sequencing depth was sufficient to reveal the bacterial diversity of the oat silage.

**Figure 1:**
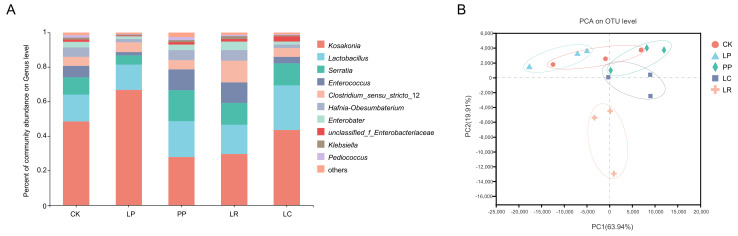
The relative abundance of microbial community at the genus level (**A**) and principal component analysis (PCA) based on operational taxonomic units (OTU) level of the oat silage after 60 days of ensiling (**B**). LP, *L. plantarum* F1; LR, *L. rhamnosus* XJJ01; LC, *L. paracasei* XJJ02; *L. paracasei*; PP, *P. acidipropionici* 1.1161.

The bacterial community based on genus-level classification is shown in Figure 1A. The dominant genera included *Kosakonia* (0.4851) and *Lactobacillus* (0.1548) in the CK group. The main genera in LP group were *Kosakonia* (0.6670) and *Lactobacillus* (0.1475). In addition, the dominant genera in PP group were *Kosakonia* (0.2785) and *Lactobacillus* (0.2069). *Kosakonia* (0.2963) and *Lactobacillus* (0.1689) were the dominant genera in LR. *Kosakonia* (0.4359) and *Lactobacillus* (0.2573) were also the dominant genera in the LC group. Principle component analysis (PCA) revealed that components 1 and 2 could explain 63.94% and 19.91% of the total variation, respectively. LR was different from the other treatment groups, and LP and CK were similar but different from the other groups (Figure 1B). It also indicates that LR was located outside the gathering point of all the groups.

### 3.3. Gas Production and In Vitro Fermentation of the Oat Silage

The effects of the inoculant treatments on gas production were largely significant within 48 h of fermentation, which indicates lower gas production in PP, LC and LR than that in LP and CK (Table 5, Figure S1). However, the rate of gas production showed no significant difference, as shown in Table 6. The theoretical maximum of gas production of in vitro fermentation of the oat silage predicted by the model was close to the actual gas production. There was no significant difference between the treatments in methane and carbon dioxide production (*p* > 0.05). In addition, the effects of the treatments on pH and AN were largely insignificant.

## 4. Discussion

The forage oat industry has been well-developed domestically, and the research on oat silage and lactic acid bacteria has attracted widespread attention. *Propionibacterium* widely exists in dairy products and silage. However, studies on the application of propionic acid bacteria in oat silage are relatively rare. Therefore, this study investigated the different effects of four additives including *propionibacterium* and common lactic acid bacteria on the fermentation quality and microbial composition of oat silages.

Higher dry matter was detected in the LR group compared to the other additives and CK, suggesting that LR has the potential to reduce silage dry matter losses. This supports the hypothesis that the substance metabolism of three lactic acid bacteria and *propionibacterium* in oat silages is different. WSC also plays a key role in the energy metabolism of microorganisms during the ensiling process. This study found that LR, LP and LC all had a better lactate synthesis capacity compared to PP and CK, which is consist with the result of the previous study [18].

The ensiling process reduces the pH through microbial anaerobic fermentation and provides a stable environment that could inhibit harmful microorganisms. As the DM content increases, the bacterial activity is restricted by low moisture. Usually, due to the lactic acid synthesis by lactic acid bacteria, the final pH of well-fermented and preserved silages could be reduced to 4.0 or lower [19,20]. In this study, the LP group had the lowest pH, which is consistent with the results of the previous studies [21,22]. LR could also reduce pH, which is consistent with Chen’s report [23]. However, unlike lactic acid, propionic acid contributed less to pH reduction [24], which may explain the higher pH in the PP group. Ammonia N is another component which is important for assessing fermentation quality. Palatability, intake and N utilization decline as the NH_3_-N content increases in silages. Fast acidification in the initial days of ensiling is essential to controlling the reproduction of clostridium bacteria, which may lead to hydrolysis of proteins and amino acids and produce large amounts of ammonia nitrogen [25]. Unlike PP, LC and CK, LP and LR could reduce the NH_3_-N content in oat, which is consist with the results of the previous studies [21,26]. Therefore, the comparison between different treatments clearly showed the benefits of inoculation under favorable ensiling conditions. This is likely to exacerbate the differences among treatments under the high DM content of the oat silage. Lactic acid is an important product in silage fermentation that reduces pH to achieve a stable state conducive to storage. Compared to PP, LC and CK, LR significantly increased the LA content, which might be attributed to the high amount of lactic acid bacteria and the WSC content, resulting in rapid production of lactic acid. Britt reported that the lower LA content in silages might be attributed to the addition of propionic acid [27]. In contrast, in this study, the PP-treated silage did not reduce the LA content probably due to the amount of lactic acid bacteria. Thus, propionic acid and *P. acidipropionici* may have different functions in oat silage. Flieg’s point, which is based on pH and DM content, has been widely used to assess the fermentation quality of silages [28]. Although additive treatments improved the fermentation quality of oat silage, only LP had a significantly increased Flieg’s point. Flieg’s point was above 50 for the treatments, which represented a moderate quality [29].

Coverage of all treatments exceeded 0.999, indicating that the sequencing data were sufficient to reflect the profile of bacterial community. In general, the stable state of aerobic and acidic environments inhibits the growth of most microorganisms, leading to low ecological diversity. PP and LC treatments led to the significant increase in the Shannon index of the oat silage, suggesting that PP and LC had a highly diverse bacterial community. Due to different treatments, index calculation methods and the number of observed OTUs, only the Shannon index showed differences. The Simpson index also indicated that PP had high bacterial community diversity. The ACE and Chao indices indicated that all treatments had the same richness of oat silage. In addition, the ACE and Chao indices may be less susceptible to inoculant treatment in oat silage, which is consist with the result of Jia’s previous study [8].

To the best of our knowledge, forage sources affect the bacterial community of silages [30]. Apparently, *Kosakonia* has been observed by many researchers in barley silage [31], stylo silage [32], ricestraw silage [33] and cornstalk silage [34]. *Kosakonia* is a genus of *enterobacteria* in the family of Enterobacteriaceae. To date, it has been identified in many studies, but no adverse effects on the ensiling process have been found. Most importantly, *Kosakonia* is considered to have the ability to reduce ammonia nitrogen [32]. Unlike LP, which may have a tendency to increase the abundance of *kosakonia* in oat silage, the addition of vanillic acid could reduce the abundance of *kosakonia* in stylo silage [35]. In addition, tannic acid could lead to the higher abundance of *kosakonia* in stylo silage [36]. *Kosakonia* is positively related to many volatile chemicals in silage [37]. In addition, the different abundances of *Clostridium sensu stricto* 12 could be related to the inhibitory ability of different strain treatments and soil contamination of the plants during harvest [38]. Despite the increasing use of high-throughput sequencing in the field of ensiling in recent years, efforts in analyzing certain unknown microorganisms, especially non-cultivable bacteria, are still expected [39]. It is difficult to draw a definitive conclusion from the relative abundance of microbial communities, so we tried to obtain more intuitive characteristics through PCA analysis. *Lacticaseibacillus rhamnosus* was thought to inhibit ethanol fermentation and *enterobacter* activity in grass silage [40]. In this study, we hypothesized that LR and other additives had slightly different metabolic patterns and expression of functional genes in the fermentation process. Moving forward, increasing studies are expected to consider not only how relative abundance of bacterial communities in the silage varieties, but also the functional genes, present.

In vitro gas production is not only applied to determine the nutritive values of silage, but is also used to predict the digestion status of ruminants [41]. In vitro gas production is highly dependent on the availability of soluble fractions, which favors ruminal fermentation at an earlier fermentation stage [42]. In this study, LP increased gas production within the first 48 h, and this might be because LP treatments could provide more available substrates for microbial degradation at the initial rumen liquid fermentation stages. However, PP and LC treatments may consume the key substrates in the ensiling process, which is good for gas production in in vitro fermentation. Intriguingly, gas production within 72 h had no significant difference, which may possibly be explained by different bacterial inoculants preferring slowly/rapidly degradable DM fractions in oat silages. Similar to Chen’s reports [41], there was no significant difference in the gas production rate constant between oat silage treated with LP and PP, which is possibly due to the rumen liquid source (ruminant species) or forage characters. Therefore, further studies should pay more attention to those different bacterial inoculants, whereas their internal biological mechanisms need to be clarified. Methane (CH_4_) is the third most important greenhouse gas, following water vapor and CO_2_, contributing to climate change [43]. Of the 16.5 billion tons of greenhouse gas emissions from global total agri-food systems in 2019, 7.2 billion tons came from the farm gate according to the new analysis [44]. The emission of methane will also cause energy loss (from 2% to 12% of gross energy (GE)) [45]. Therefore, significant research investments are needed to reduce the carbon footprint of ruminants, improve rumen fermentation and clarify the proton transfer strategy [46]. Although the in vitro gas production (both methane and carbon dioxide) of oat silage fermented with PP and LC showed no significant difference from other groups, both reduced the emissions of methane and carbon dioxide. We hypothesized that both PP and LC might have positive effects on rumen methane proton transfer. Both oat grain and whole plants contain several types of antioxidants, such as α-tocopherol [47] and polyphenols [48]. *Propionibacterium acidipropionici* 1.1161. and *Lacticaseibacillus paracasei* treatments may protect the antioxidants in oat ensiling processing [49], thereby regulating the metabolism of rumen methane bacteria and reducing gas production. Moreover, those additives may provide a new prospect for oat silage fermentation and digestion and utilization of ruminants.

## 5. Conclusions

The ability of *L. plantarum* F1 to improve the quality of oat silages was stronger than that of *P. acidipropionici* 1.1161, even *L. rhamnosus* XJJ01 and *L. paracasei* XJJ02. At the same time, *L. plantarum* F1 reduced the bacterial community diversity in the ensiling process and caused the oat silage to produce more gas at an earlier in vitro fermentation stage. Compared to other additives, *L. paracasei* XJJ02 and *P. acidipropionici* 1.1161 may result in less greenhouse gas production in rumens. Consequently, this finding could help us to choose more suitable silage additives for different feeding strategies.

## Figures and Tables

**Table 1:** Chemical composition and microbial population of oat by plate culture before ensiling.

Items	Forage Oat
Dry matter g kg^−1^ FM	354.7
Water-soluble carbohydrates g kg^−1^ DM	16.3
Crude protein g kg^−1^ DM	79.0
Neutral detergent fiber g kg^−1^ DM	461.8
Acid detergent fiber g kg^−1^ DM	259.2
Ether extract g kg^−1^ DM	61.6
pH	6.43
Lactic acid bacteria (log cfu g^−1^ FM)	4.74
Coliform bacteria (log cfu g^−1^ FM)	7.28
Yeast (log cfu g^−1^ FM)	6.60

FM, fresh matter; DM, dry matter; cfu, colony-forming units.

**Table 2:** Chemical composition and microbial population of the oat silage by plate culture.

Items	Treatments						
	CK	LP	PP	LC	LR	SEM	*p*-Value
Dry matter g kg^−1^ FM	335.5 b	335.6 b	347.1 b	336.9 b	363.3 a	0.006	0.004
Crude protein g kg^−1^ DM	81.1	78.6	86.1	81.5	80.3	0.213	0.053
Ether extract g kg^−1^ DM	51.3	48.6	47.0	46.9	54.3	0.004	0.327
Neutral detergent fiber g kg^−1^ DM	521.6	508.8	516.6	507.8	503.8	1.248	0.627
Acid detergent fiber g kg^−1^ DM	296.7	296.9	296.	293.9	285.0	0.836	0.598
Water-soluble carbohydrates g kg^−1^ DM	5.1 c	7.9 ab	6.3 bc	9.0 a	8.4 ab	0.106	0.024
Yeast log cfu g^−1^ FM	-	-	3.0	-	-	-	-
Coliform bacteria log cfu g^−1^ FM	-	-	-	-	3.3	-	-
Lactic acid bacteria log cfu g^−1^ FM	6.83 b	6.18 c	7.22 b	6.65 bc	8.02 a	0.274	0.001

LP, *L. plantarum* F1; LR, *L. rhamnosus* XJJ01; LC, *L. paracasei* XJJ02; *L. paracasei*; PP, *P. acidipropionici* 1.1161; SEM, standard error of the mean; FM, fresh matter; LAB, lactic acid bacteria; cfu, colony-forming units; values with different letters in the same row are significantly different (*p* < 0.05).

**Table 3:** Fermentation quality of the oat silage.

Items	CK	LP	PP	LC	LR	SEM	*p*-Value
pH	4.09 ab	3.90 c	4.20 a	4.16 a	3.97 bc	0.073	0.010
NH_3_-N g kg^−1^ TN	58.4 a	39.3 b	57.0 a	51.0 a	57.4 a	0.471	0.011
LA g kg^−1^ DM	50.0 b	57.9 ab	50.5 b	45.5 b	63.5 a	0.534	0.046
AA g kg^−1^ DM	6.4	6.9	6.0	4.3	4.0	0.227	0.635
PA g kg^−1^ DM	6.7	6.8	9.1	6.8	7.3	0.124	0.327
BA g kg^−1^ DM	4.8 a	2.5 b	4.6 ab	2.5 b	4.90 a	0.084	0.036
Flieg’s point	57.00 b	75.00 a	59.00 b	66.67 ab	62.50 b	4.415	0.019

LP, *L. plantarum* F1; LR, *L. rhamnosus* XJJ01; LC, *L. paracasei* XJJ02; *L. paracasei*; PP, *P. acidipropionici* 1.1161; SEM, standard error of the mean; TN, total nitrogen; AN, ammonia nitrogen; DM, dry matter; LA, lactic acids; AA, acetic acids; PA, propionic acids; BA, butyric acids; values with different letters in the same row are significantly different (*p* < 0.05).

**Table 4:** Estimation of bacterial community diversity of the oat silage.

Index	CK	LP	PP	LC	LR	SEM	*p*-Value
Shannon	1.69 ab	1.19 b	2.11 a	2.03 a	1.68 ab	0.265	0.042
Simpson	0.32	0.49	0.17	0.20	0.28	0.108	0.085
ACE	30.54	29.57	32.54	32.32	30.16	2.571	0.711
Chao	30.17	28.67	32.67	31.67	29.5	2.519	0.536
Coverage	0.999	0.999	0.999	0.999	0.999	<0.0001	0.923

LP, *L. plantarum* F1; LR, *L. rhamnosus* XJJ01; LC, *L. paracasei* XJJ02; *L. paracasei*; PP, *P. acidipropionici* 1.1161; SEM, standard error of the mean; values with different letters in the same row are significantly different (*p* < 0.05).

**Table 5:** Cumulative gas production (mL/200 mg DM) of the oat silage within 72 h of in vitro fermentation.

Time	CK	LP	PP	LC	LR	SEM	*p*-Value
2 h	12.50 b	16.42 a	9.13 c	10.25 bc	11.42 bc	1.401	0.001
4 h	15.09 b	19.09 a	12.67 bc	12.25 c	13.92 bc	1.153	<0.001
6 h	18.17 b	22.25 a	15.63 bc	13.58 c	17.25 b	1.488	<0.001
8 h	21.17 b	25.75 a	17.42 c	17.59 c	19.34 bc	1.418	<0.001
12 h	24.58 b	28.48 a	20.96 c	21.08 c	23.00 bc	1.401	<0.001
24 h	27.70 ab	31.50 a	22.00 b	21.50 b	24.60 b	2.710	0.016
36 h	30.12 ab	35.62 a	24.25 b	22.32 b	29.00 ab	3.793	0.048
48 h	32.58 ab	38.58 a	25.88 b	23.85 b	30.00 ab	4.191	0.049
72 h	32.92 ab	39.75 a	27.63 b	26.63 b	32.63 ab	4.430	0.108

LP, *L. plantarum* F1; LR, *L. rhamnosus* XJJ01; LC, *L. paracasei* XJJ02; *L. paracasei*; PP, *P. acidipropionici* 1.1161; SEM, standard error of the mean; values at different time points with different letters in the same row are significantly different (*p* < 0.05).

**Table 6:** In vitro fermentation characteristics and greenhouse gas production (72 h) of the oat silage.

Item	CK	LP	PP	LC	LR	SEM	*p*-Value
B (mL 200 mg^−1^ DM)	31.15	36.50	25.13	25.66	30.12	4.895	0.104
c (mL h^−1^)	0.15	0.20	0.17	0.14	0.17	0.048	0.910
pH	6.69	6.65	6.71	6.64	6.67	0.036	0.452
NH_3_-N (mmol L^−1^)	22.10	22.09	22.22	22.22	22.10	0.073	0.222
Methane mL	6.19	8.39	5.45	5.79	7.29	1.290	0.347
Carbon dioxide mL	19.21	21.46	15.75	15.26	19.34	3.420	0.507
Others mL	3.86	7.16	4.04	6.04	6.00	1.636	0.44

LP, *L. plantarum* F1; LR, *L. rhamnosus* XJJ01; LC, *L. paracasei* XJJ02; *L. paracasei*; PP, *P. acidipropionici* 1.1161; SEM, standard error of the mean; AN, ammonia nitrogen; B, the potential gas production; c, the gas production rate constant.

## Data Availability

The data presented in this study are available in this article (and Supplementary Material).

## Supplementary Materials

The following supporting information can be downloaded at: https://www.mdpi.com/article/10.3390/ani12091122/s1, Figure S1: Dynamic gas production from oat silage during 72 h of in vitro fermentation.
