# Supplementary material for: Exploring the Effects of Different Bacteria Additives on Fermentation Quality, Microbial Community and In Vitro Gas Production of Forage Oat Silage
Source: Animals (Basel). 2022 Apr 27;12(9):1122. doi: 10.3390/ani12091122 (PMC9100174; doi:10.3390/ani12091122)
Supplement: Supplementary file 1 [file animals-12-01122-s001.zip › animals-1660144-supplementary.pdf]

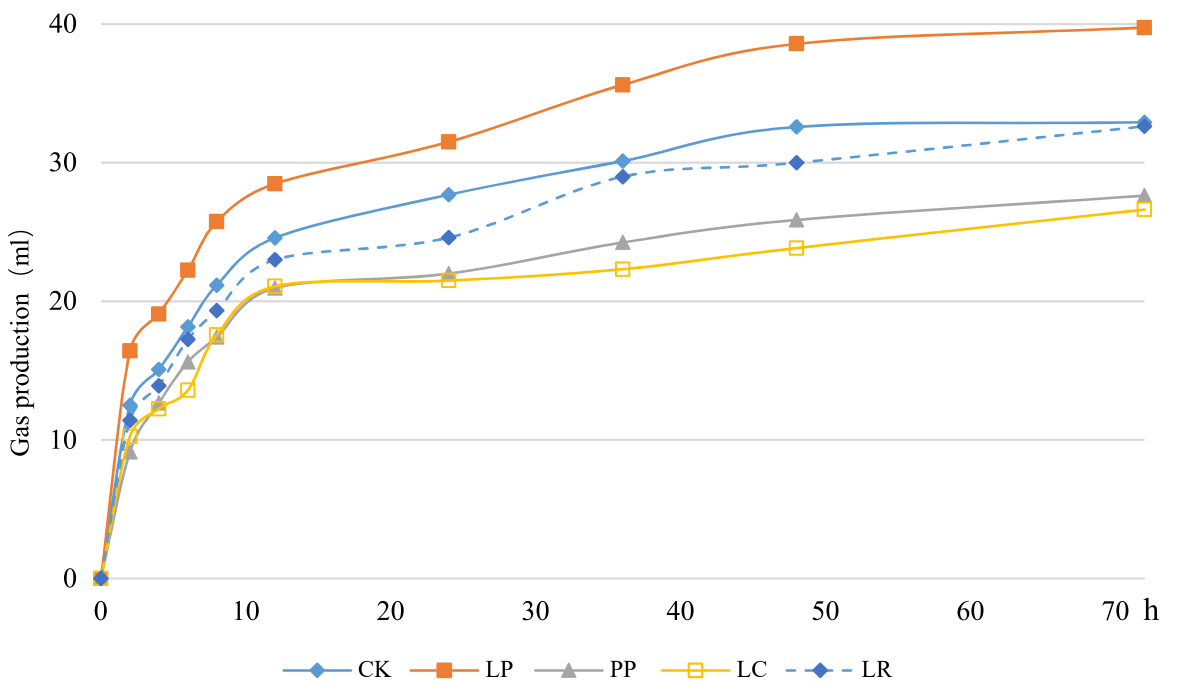

Figure S1 Dynamic gas production from oat silage during 72 hours of in vitro fermentation

LP, *L. plantarum* F1; LR, *L. rhamnosus* XJJ01; LC, *L. paracasei* XJJ02; *L. paracasei*; PP, *P. acidipropionici* 1.1161;
